# The Function of Probiotics and Prebiotics on Canine Intestinal Health and Their Evaluation Criteria

**DOI:** 10.3390/microorganisms12061248

**Published:** 2024-06-20

**Authors:** Junliang Xia, Yuling Cui, Yan Guo, Yuwen Liu, Baichuan Deng, Sufang Han

**Affiliations:** Guangdong Provincial Key Laboratory of Animal Nutrition Control, College of Animal Science, South China Agricultural University, Guangzhou 510642, China; xjl465181167@126.com (J.X.); 13794095419@163.com (Y.C.); gy@scau.edu.cn (Y.G.); liu.evan0319@foxmail.com (Y.L.)

**Keywords:** probiotics, prebiotics, intestinal microbiota, canine intestinal health, intestinal diseases, evaluation criteria

## Abstract

Maintaining homeostasis within the intestinal microbiota is imperative for assessing the health status of hosts, and dysbiosis within the intestinal microbiota is closely associated with canine intestinal diseases. In recent decades, the modulation of canine intestinal health through probiotics and prebiotics has emerged as a prominent area of investigation. Evidence indicates that probiotics and prebiotics play pivotal roles in regulating intestinal health by modulating the intestinal microbiota, fortifying the epithelial barrier, and enhancing intestinal immunity. This review consolidates literature on using probiotics and prebiotics for regulating microbiota homeostasis in canines, thereby furnishing references for prospective studies and formulating evaluation criteria.

## 1. Introduction

Dogs are one of the most important companion animals, and their intestinal health has become a research focus. The abundance and diversity of canine intestinal microbiota increase gradually along the gastrointestinal tract [1]. Due to differences in the anatomy and physiology of the canine gastrointestinal tract, there are variations in the microbial populations among the stomach, small intestine, and large intestine [2]. The stomach harbors only a limited number of bacteria that can survive the acidic environment, mainly *Helicobacter* spp. and lactic acid bacteria [3]. Both aerobes and anaerobes coexist in the small intestine [4]. The large intestine contains a high population of anaerobic bacteria [5]. The canine intestinal tract is dominated by *Firmicutes*, *Bacteroidetes*, *Proteobacteria*, and *Actinobacteria*, although their abundance and diversity vary widely among individuals. *Lactobacillus* is also widely distributed within the Firmicutes in the canine intestine, with a typical count of 10^4^–10^8^ CFU/mL [6]. Current studies have demonstrated that canine intestinal microbiota undergo significant changes when affected by inflammatory bowel disease (IBD), acute hemorrhagic diarrhea (AHD), acute diarrhea (AD), and other gastrointestinal diseases. For example, the number of *Salmonella*, *Sutterella*, *Escherichia coli, Actinomycetes, Erysipelas*, and *Clostridium perfringens* increases, while *Lactobacillus* decreases [7,8,9,10,11]. These studies indicate a strong relationship between canine gastrointestinal diseases and intestinal microbiota dysbiosis. Therefore, regulating intestinal microbiota homeostasis has been recognized as an effective way to maintain canine intestinal health, improve intestinal immunity, and promote canine well-being [6,12]. Probiotics and prebiotics have been used to treat AD, ulcerative colitis (UC), and irritable bowel syndrome (IBS) in humans, and a clear standard has been gradually established [13,14,15].

Although numerous prebiotic and probiotic products targeting canine health are available, a unified evaluation standard system needs to be present. This deficiency significantly impedes the advancement and utilization of probiotics and prebiotics. This review aims to undertake a comparative analysis of various clinical trials and applications of probiotics and prebiotics in canines, aiming to delineate an objective evaluation framework and discussion to support future studies of probiotics and prebiotics. 

## 2. Probiotics and Prebiotics

The Food and Agriculture Organization of the United Nations/World Health Organization (FAO/WHO) defines probiotics as active microorganisms that are beneficial to host health when consumed in sufficient quantities [16]. Currently, it has been reported that more than ten genera of microorganisms, including *Propionibacterium*, *Peptostreptococcus*, *Lactobacillus*, *Bacillus*, *Enterococcus*, *Bacteroides*, *Streptococcus*, *Lactococcus*, *Bifidobacterium*, *Akkermansia*, *Saccharomyces*, and *Pediococcus*, are regarded as potential probiotics to treat chronic diseases like IBD and diabetes [15]. According to the current “Catalogue of Feed Additives” in China, twenty-five species of bacteria, including *Lactobacillus fermentans*, *Bifidobacterium animalis*, and *Enterococcus faecalis*, and five species of fungi, including *Saccharomyces cerevisiae* and *Candida prion-producing yeast*, are approved for use in cats and dogs (Announcement No. 744 of the Ministry of Agriculture and Rural Affairs of China). 

In 2016, the expert panel of the International Scientific Association for Probiotics and Prebiotics (ISAPP) revised the definition of prebiotics as “a substrate that is selectively utilized by host microorganisms conferring a health benefit”. This expanded definition includes noncarbohydrate or other nonfood substances that can be applied to body parts beyond the gastrointestinal tract [17,18]. According to Gibson et al. [19], a substrate cannot be referred to as a prebiotic if it produces adverse effects via host intestinal microbiota utilization. 

Prebiotics are widely derived from natural sources, such as fructooligosaccharides (e.g., onion, leek, wheat, and chicory) [20], isomalto-oligosaccharides (soy, sauce, sake, and honey) [21], galacto-oligosaccharides (e.g., lentil, green pea, lima bean, and kidney bean) [22], and inulin (e.g., agave, banana/plantain, and burdock camas) [23]. According to the current “Catalogue of Feed Additives” in China, prebiotics used in cats and dogs mainly consist of fructo-oligosaccharides, manno-oligosaccharides, galacto-oligosaccharides, and other polysaccharides (Announcement No. 744 of the Ministry of Agriculture and Rural Affairs of China).

## 3. The Function of Probiotics and Prebiotics on Canine Intestinal Health

The intestinal microbiota is proposed to be involved in digestion, immunity, and other biological processes [24]. Probiotics have been demonstrated to regulate intestinal health via multiple mechanisms [25]. Probiotics and prebiotics improve intestinal health primarily by interacting with the intestinal microbiota, increasing beneficial intestinal metabolites, enhancing mucosal barrier properties, and promoting cellular and humoral immunity [25,26,27]. For example, some lactic acid bacteria can increase the concentration of organic acids in the intestinal tract to inhibit the survival of pathogenic bacteria, and it has also been reported that these organic acids enhance immune function and improve inflammation [28,29]. The following article will elaborate on the aspects of probiotic and prebiotic products in maintaining canine intestinal health and how to evaluate these products more comprehensively and accurately.

### 3.1. Interaction with Intestinal Microbiota

**Figure 1:**
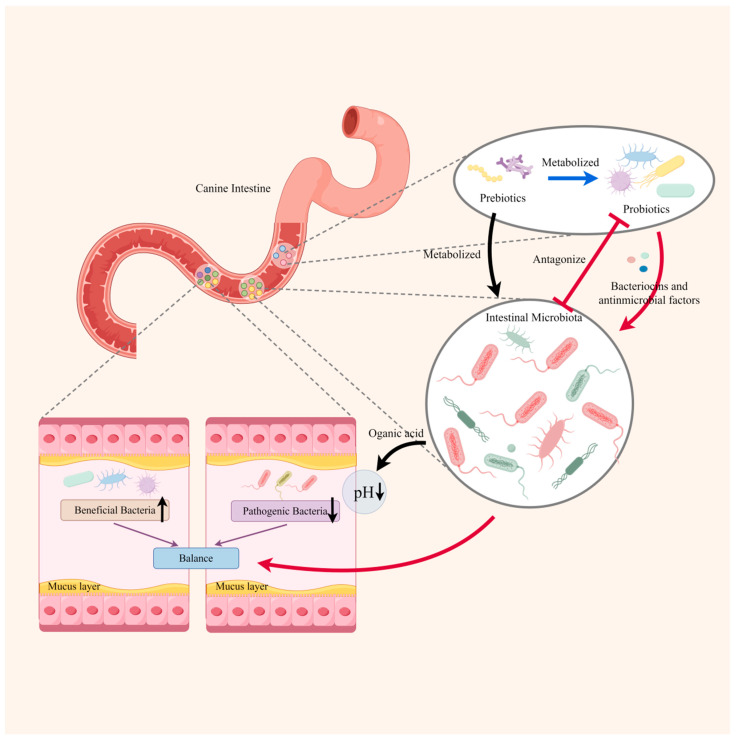
The interaction between intestinal microbiota and probiotics and prebiotics (by figdraw). The interaction between probiotics and intestinal microbiota tends to be antagonistic. Probiotics will compete with the intestinal flora for nutrients and inhibit the colonization of the intestinal microbiota through secreting antimicrobial peptides and bacteriocins. On the other hand, probiotics or intestinal microbiota will metabolize the prebiotics to produce organic acids (e.g., short-chain fatty acids and lactic acid), which lower the pH in the intestinal tract. Eventually, the number of beneficial bacteria increases, the number of pathogenic bacteria decreases, and the intestinal microecology reaches balance with the action of low pH value and substances such as antimicrobial peptides and bacteriocins.

Probiotics can interact with the intestinal microbiota through competition for nutrients, antagonism, or secretion of bacteriocins and other antimicrobial factors to support microbiota stability [30]. Recent research indicated that probiotics can inhibit the colonization of pathogens, improve microbial diversity, and restore intestinal microbiota homeostasis by resisting the colonization of pathogens and increasing the mass of probiotics [31,32,33,34]. Figure 1 shows the interaction between probiotics and intestinal microbiota. For example, supplementing canines suffering from AD with probiotics resulted in a lower number of pathogenic bacteria in their feces, such as *Escherichia coli*, *Clostridium perfringens*, *Fusobacterium*, and *Dantobacter*, compared to the placebo group [35,36,37]. Another trial conducted on healthy dogs showed that adding a suitable amount of specific probiotics (*Bacillus amyloliquefaciens* CECT5940 and *Enterococcus faecium* CECT4515) to their diet helped to regulate the homeostasis of intestinal microbiota by promoting the growth of lactic acid bacteria and inhibiting the proliferation of pathogenic bacteria [38]. In addition, using probiotics instead of antibiotics to regulate the intestinal microbiota of dogs is also one of the current research hotspots. A variety of *Lactobacillus* have killing effects on some common therapeutic bacteria, such as *Escherichia coli*, *Staphylococcus aureus*, and *Salmonella enteritidis* [39,40,41]. However, there are still few reports on animal experiments. In addition, we need more clinical experimental data on treating diseases caused by pathogenic bacteria to evaluate the antibacterial effect of probiotics. The host intestinal microbiota can metabolize some inactivated probiotics to produce teichoic acid, organic acids, peptides, and other substances. These substances can promote host digestion and absorption, balance the intestinal microbiota, and protect the intestinal mucosa [6,42,43]. 

Prebiotics, such as mannose-oligosaccharides (MOS), fructo-oligosaccharides (FOS), and galacto-oligosaccharides (GOS), play a vital role in balancing intestinal microbiota. For instance, adding FOS to the canine diet increased the number of probiotic bacteria, including *Bifidobacterium* and *Lactobacillus*, while reducing the number of *Clostridium perfringens* [44,45]. Clinical trials in other mammals indicated that prebiotics, such as fructo-oligosaccharides, galacto-oligosaccharides, and pectin oligosaccharides, can effectively promote the proliferation of lactic acid bacteria in the intestine [46,47]. 

Changes in the genus and abundance of canine intestinal microbiota are regarded as a preferable measure of the effect of probiotics on intestinal health [24]. At present, the technology for detecting intestinal microbiota is gradually maturing. For instance, 16S rRNA sequencing can determine the changes in the microbiota at the genus level, and metagenomic sequencing can even analyze the intestinal microbiota at the species level. Due to the high cost, deep whole-metagenome shotgun sequencing was hard to apply to companion animals [2]. However, it has been reported that shallow shotgun sequencing is a possible cost-effective alternative to 16S rRNA sequencing for large-scale biomarker discovery with improved taxonomic resolution and functional accuracy [48]. Moreover, qPCR and FISH hybridization techniques can detect specific bacteria. However, the drawback is that it is difficult to cope with the large number of bacteria in the intestine [2]. Therefore, it is necessary to use a variety of omics sequencing, such as shallow shotgun metagenomics and 16S rRNA sequencing, to evaluate the effects of probiotics and prebiotics on the health of canine intestinal microbiota. At the same time, it is also necessary to combine metabolomics or proteomics to detect and analyze the key metabolites (such as SCFAs) or enzymes of canine intestinal microbiota [34].

### 3.2. Improving Intestinal Barrier Function

The intestinal mucosal epithelium serves as the primary line of defense against pathogens and is essential in stimulating adaptive immune signals [49]. Probiotics can improve the microenvironment of intestinal epithelial cells and boost the resistance of the intestinal mucosa to pathogenic bacteria by stimulating immune cells to secrete substances, such as lysozyme, secretory phospholipase A2, and defensin [50,51]. Probiotics also play a vital role in reducing inflammation by decreasing epithelial cell apoptosis, increasing the number of anti-inflammatory bacteria (e.g., *Butyricimonas* and *Prevotella*), and maintaining intestinal barrier function [52,53,54,55]. Another way in which probiotic strains may improve barrier function is through probiotic-derived proteins, which can transactivate Epidermal Growth Factor Receptor signaling to ameliorate cytokine-induced apoptosis and mitigate disruption of the epithelial barrier and inflammation [52,56]. Improving the intestinal barrier function may be a potential indicator for evaluating the probiotic strain since not all can improve barrier function [34].

Prebiotics also have anti-viscosity properties that can bind to receptors on intestinal mucosal epithelial cells. This capability can inhibit pathogens from attaching to glycoproteins on the cell surface, reducing the probability of pathogen adhesion and invasion while enhancing the barrier function of the intestinal mucosa [47,57].

### 3.3. Enhancing the Cell-Mediated Immune and Humoral Immune Function

**Figure 2:**
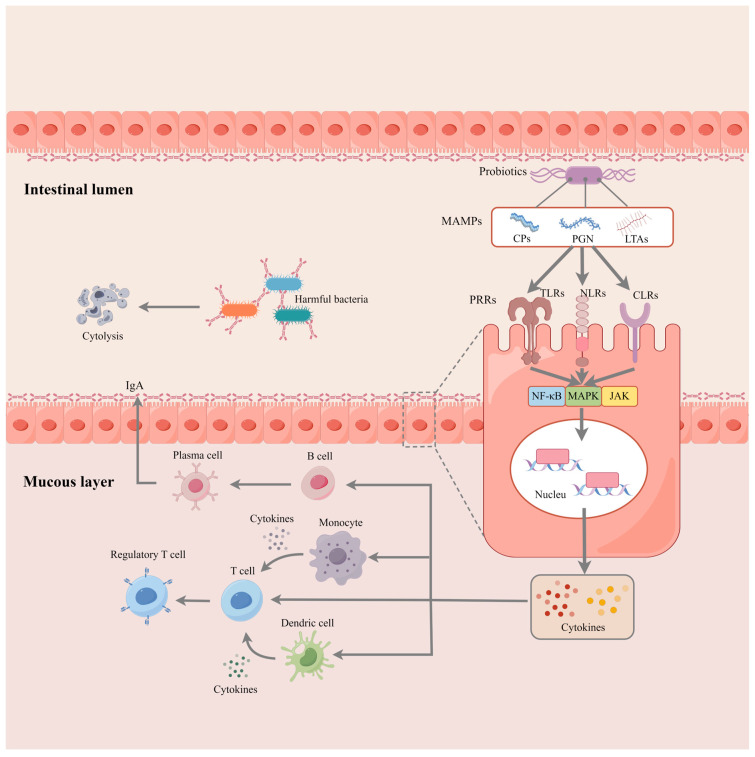
Activation of intestinal immunology via probiotics and prebiotics (by figdraw). The MAMPs of probiotics can be recognized via PPRs expressed on the epithelial cell membrane, thereby activating the transcription of cytokines through signaling pathways such as NF-κB. Cytokines produced by intestinal epithelial cells can trigger the native immune response in the mucous layer. For instance, this molecule can transactivate monocytes and dendritic cells to secrete chemokines and other cytokines (e.g., TNF) that further promote the proliferation and differentiation of T lymphocytes. Moreover, B cells rapidly respond to these signals by differentiating into plasma cells and secreting antibodies (e.g., IgA) into the intestinal lumen. The IgA forms a line of defense on epithelial cells and binds to receptors on the surface of pathogenic bacteria, promoting bacterial lysis and clearance.

Figure 2 illustrates the role of probiotics in activating innate immunity. Probiotics possess conserved microbe-associated molecular patterns (MAMPs) [including cell wall polysaccharides (CPs), peptidoglycan (PGN), lipoprotein anchors, and lipoteichoic acids (LTAs)], which can interact with pattern recognition receptors (PRRs) [including the Toll-like receptors (TLRs), C-type lectin receptors (CLRs), and nucleotide oligomerization domain (NOD)-like receptors (NLRs)]. PRRs are broadly expressed in various cell types, including epithelial and immune cells. Thus, epithelial cells, dendritic cells, and monocytes can recognize probiotics or their molecules through pattern recognition receptors such as Toll-like receptors (TLRs), NOD-like receptors (NLRs), and C-type lectin receptors. This recognition triggers a signaling pathway (e.g., NF-κB and MAPK) that promotes the secretion of immune factors such as interleukins, tumor necrosis factors, interferons, transforming growth factors, and chemokines [58,59,60,61]. These cytokines build a regulatory network, activate the innate immune cells, and promote their differentiation and function [62,63]. In a mouse model, Liu et al. [64] found that *L. casei* M2S01 alleviated the symptoms of IBD by activating the T-regs and upregulating the levels of IL-10. Another trial in mice with IBD provides evidence of the generation of CD4^+^ CD5^+^ Foxp3^+^ T-regs in response to probiotic mixtures. The upregulated chemokines (CCL1 and CCL22) then recruit CD4^+^ Foxp3^+^ Tregs to the inflammatory sites and inhibit the progression of inflammation [65].

Mammals’ intestinal lumen contains secretory IgA (sIgA), which shields the epithelium of the intestine from enteric pathogens and the toxins they produce [24]. Probiotics have been reported to enhance the intestinal barrier by promoting the production of sIgA [24,66]. Trials on dogs also indicated that probiotics can significantly increase the intestinal sIgA and plasma IgG levels, with effects lasting from weaning to one year of age [67,68]. Another trial shows that prebiotics benefit IgE and IgA secretion and enhance intestinal barrier function [69,70]. Interestingly, in addition to increasing intestinal immunoglobulins, probiotics can promote the IgG, IgM, and IgA levels in the colostrum, thereby improving neonatal clinical conditions and immune function [71].

### 3.4. Producing Beneficial Fermentation Production

**Figure 3:**
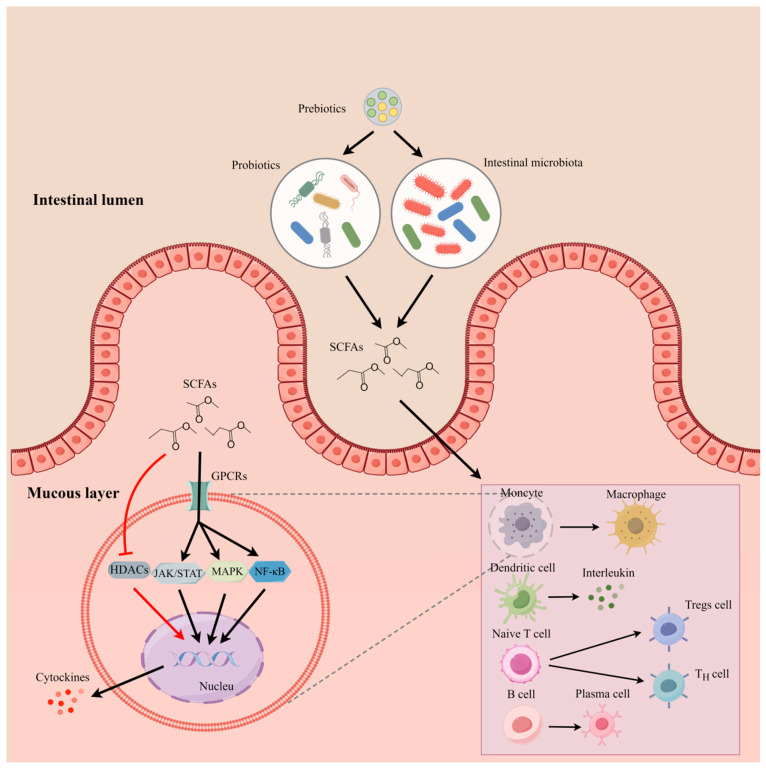
Modulation of intestinal mucous immune function by SCFAs produced by probiotics and prebiotics (by figdraw). Probiotics and intestinal microorganisms metabolize dietary fibers, such as prebiotics, to produce SCFAs, which pass through the intestinal epithelial layer into the mucous layer and modulate the function of innate immune cells. For instance, SCFAs can activate monocytes and dendritic cells to enhance their phagocytosis and secretion, promote the differentiation of T-lymphocytes to regulatory T cells and helper T cells, and promote the proliferation and differentiation of B-lymphocytes. In addition, SCFAs can bind to GPCRs and mediate the transcription and translation of cytokines (e.g., IL-12) through activating pathways such as JAK/STAT, NF-κB, and MAPK. On the other hand, SCFAs also act as inhibitors of HDACs to enhance the expression of immune factors.

Probiotic species belonging to the *Lactobacillus* and *Bifidobacterium* genera produce lactic and acetic acid to keep a lower luminal pH and discourage the growth of pathogens [34,72]. In addition to acetic acid, other beneficial short-chain fatty acids such as butyrate and propionate will also be increased under the action of probiotics and prebiotics [73,74]. Figure 3 explains how the probiotics and prebiotics regulate the innate immune response by producing SCFAs. At present, supplementation with yeast products or mannan-oligosaccharides can increase the concentration of SCFAs in the canine intestine [75,76,77]. SCFAs can regulate immune cells and intestinal inflammation through GPCRs signaling and inhibitory effects on HDACs. SCFAs can regulate T cell differentiation and function [28,29,78], promote B cell differentiation and intestinal IgA responses [79], and induce antimicrobial activity and phagocytic activity of macrophages. Moreover, SCFAs can regulate the cytokines (e.g., IL-12 and TNF) and modify cellular metabolism through JAK/STAT signaling [80]. The level of SCFAs is regarded as a predictor of some diseases [81]. Therefore, the level of SCFAs will be a valuable predictor for evaluating the effect caused by probiotic or prebiotic products.

### 3.5. Comparison of Clinical Therapy

Single probiotic strains, multiple probiotic strains, and probiotics with antibiotic treatment are the main treatments in clinical trials [82,83,84]. Improvement of clinical conditions is one of the crucial criteria for evaluating probiotic and prebiotic products, commonly including the canine chronic enteropathy clinical activity index (CCEAI), the canine IBD activity index (CIBDAI), the canine hemorrhagic diarrhea severity index (CHDSI), and intestinal histology scores. Previous studies showed that probiotic and prebiotic products can significantly improve the clinical condition, indicating these products had potential treatment effects [82,84]. However, recent reports indicate that the use of probiotic products is not clinically significant for preventing or treating acute diarrhea in dogs [85,86]. The conclusions are controversial due to the lack of uniform evaluation criteria for all the study cases compared to their reports. Thus, the therapeutic efficacy of probiotic or prebiotic products need to be evaluated in conjunction with other clinical data.

Table 1 compares the different probiotic strains on intestinal disease treatment considering four aspects: the influence on intestinal microbiota, immune and barrier function, clinical characterization, and metabolism. Previous studies have shown that many intestinal diseases are associated with dysbiosis of the intestinal microbiota [36,82]. Past research indicates that probiotics and prebiotics can influence the intestinal microbiota. However, most of the research lacked completeness, by using qPCR for specific bacteria or exploring the effects of probiotics and prebiotics on the intestinal microbiota only in terms of phylum [35,87,88]. Sequencing technology is now widely used in the study of microbiomes. Using 16SRNA sequencing or even whole genome birdshot to evaluate the clinical therapeutic efficacy of probiotic and prebiotic products on the intestinal microbiome will be more comprehensive [2]. 

Additionally, metabolites of intestinal microorganisms (e.g., SCFAs) should also be included in the criteria for evaluating the therapeutic efficacy of probiotics and prebiotics because one of the most important ways for probiotics and prebiotics to modulate the intestinal immune function of the host is through the regulation of the metabolites of the intestinal microbiota [95]. Metabolites in the blood are essential indicators of the safety of probiotics and prebiotics products, as they reflect whether the organism is healthy [37,96]. The influence of probiotic and prebiotic products on intestinal mucosal immunity is also one of the effective criteria for evaluating their effects. However, due to the limitations of the experimental subjects and experimental conditions, it is impractical to take biopsy samples from every clinically treated dog [24,66,93]. Therefore, it is possible to try to evaluate the modulatory effects of probiotics and prebiotics on the immune function of the intestinal mucosa, such as on the proliferation and differentiation of immune cells as well as on the production and secretion of immunoglobulins, with the help of other mammalian small mammal models, such as mice [93]. Some interesting issues deserve to be further explored, such as the dosage and duration of probiotic and prebiotic products for the treatment of diseases, as these conditions are also considered to be important factors influencing the therapeutic effect [97,98,99].

## 4. Prospect

Maintaining intestinal health, promoting food digestion and absorption, enhancing the intestinal barrier, and strengthening the immune system depends on intestinal microbial homeostasis [100]. Probiotic and prebiotic products mainly improve intestinal health through interacting with intestinal microbiota, improving the intestinal mucosal barrier, and enhancing intestinal immune function. A growing body of research suggests that probiotics and prebiotics are also effective alternatives to antibiotics, and there has been a gradual increase in the number of probiotic and prebiotic products on the market for use in dogs [25]. Although more and more strains with probiotic properties are being isolated, the lack of validated and harmonized evaluation criteria reported by these researchers has greatly limited the development and application of probiotic and prebiotic products in dogs [92,94].

### 4.1. The Evaluation Criteria of Probiotics and Prebiotics

The evaluation of probiotic and prebiotic products should be based on the exact dosage, duration of use, and its effect. Previous research has indicated that the effect of probiotics and prebiotics can be divided into three categories: influence on intestinal microbiota, metabolism, and clinical characterization [25,26,27,35,88]. The changes in the species of intestinal microbiota and the proportion of beneficial and harmful bacteria may reflect the effect of probiotics and prebiotics on intestinal health [7,8,9,10,11]. Hematological and serum biochemical indicators, as well as some inflammatory and immune-related indicators, such as the level of SCFAs, IgA, etc., can be used to evaluate the effect of probiotics and prebiotics on metabolism [24,66,81]. The effect of probiotics and prebiotics on clinical characterization requires appropriate evaluation criteria such as CCEAI, CIBDAI, etc., for intestinal inflammation or diarrhea-like diseases [92,93]. Therefore, qualified probiotic and prebiotic products should improve body health in these three aspects. However, it is necessary to rely on more clinical trials and data to determine the optimal amount and duration of use. For example, the Probiotics and Prebiotics Working Group of the European Society for Paediatric Gastroenterology, Hepatology, and Nutrition (ESPGHAN) has published guidelines on the use of probiotics and prebiotics after evaluating the efficacy of certain probiotics in the treatment of acute gastroenteritis in children [101,102]. Therefore, further standards for probiotic and prebiotic products in the pet industry need to be established. In addition, given the multitude of variables that influence the efficacy of these products, including breed, age, sex, body weight, diet, medical history, and antibiotic interventions, it is optimal to conduct research on the same probiotic and prebiotic products to address these factor [25].

### 4.2. Evaluating the Functions of Probiotics and Prebiotics from Multiple Dimensions

With the development of microbiome sequencing, the improvement of sequencing technology and the reduction in cost will make it easier for researchers to comprehensively assess the effects of probiotics and prebiotics on canine intestinal microbiota at the species level using 16SRNA sequencing or even whole-genome birdshot, revealing the interactions between various potential probiotic strains and prebiotics with the intestinal microbiota, and providing a basis for screening effective probiotics and prebiotics [2,34]. In addition, the existing histologic databases for dogs of different breeds and regional origins still need to be occupied, which makes it challenging to establish microbiota criteria for intestinal health and is not conducive to research. In addition, the effect of probiotic and prebiotic products on the metabolic effects of canine intestinal microflora is also an important aspect. For instance, SCFAs are important for canine intestinal immunity. However, previous studies have only elucidated the mechanism of SCFAs on intestinal immune cells and mucosal function in other animal models, such as mice, and their mechanism of action in the canine has not yet been revealed [80]. In addition to SCFAs, other metabolites, such as lactic acid and some enzymes, antimicrobial peptides, bacteriocins, and the effects of these metabolites on intestinal health need to be further investigated and verified [34]. Combining the genomics and metabolomics of intestinal microorganisms to analyze the role of probiotics and prebiotics comprehensively may be the way forward.

## 5. Conclusions

Using probiotic and prebiotic products is an effective strategy for improving canine gut health. Probiotic and prebiotic products can improve canine intestinal health in several ways, including balancing the intestinal microbiota, regulating intestinal immune function, improving inflammation, enhancing intestinal mucosal barrier function, and modulating intestinal metabolites. In this paper, we review the research and application cases of probiotics and prebiotics in dogs and propose an evaluation system to assess the effects of probiotics and prebiotics in terms of the influence of intestinal microbiota, improvement of clinical diseases, intestinal metabolites, intestinal immune function, and barrier function. In addition, multi-omics analysis and screening of some critical metabolic or immune indicators will help to develop new probiotic and prebiotic products and better evaluate the effectiveness of probiotics and prebiotics.

## Figures and Tables

**Table 1:** Probiotics commonly used to treat canine diseases.

Types of Diseases	No. of Dogs	Treatment Duration	Probiotics	Influence on Intestinal Microbiota	Immune and Barrier Function	Clinical Characterization	Metabolism	Reference
Acute diarrhea (AD)	31	14 days	*Bifidobacterium animalis* *strain AHC7*	No data.	No data.	Treated group shortened the duration of diarrhea (3.9 ± 2.3 days vs. 6.6 ± 2.7 days; *p* < 0.01).	No data.	[83]
Acute diarrhea (AD)	733	28 days	*Enterococcus faecium* (NCIMB10415 SF68)	No data.	No data.	Shortened the diarrhea days and significantly decreased the diarrhea incidence.	No data.	[89]
Acute diarrhea (AD)	148	10 days	*E. faecium* 4b1707	No data.	No data.	Shortened the duration of diarrhea.	No data.	[90]
Chronic enteropathies (CE)	12	42 days	*Enterococcus faecium* (NCIMB10415 SF68)	No data.	There were no significant differences in intestinal immune gene expression.	There were no significant differences in CCEAI and histology scores.	There were no significant differences in hematological and biochemical variables.	[91]
Chronic enteropathies (CE)	20	60 days	*Saccharomyces boulardii*	No data.	No data.	Significantly reduced stool frequency and improved the stool consistency. Significantly increased the body condition score (BCS).	There were no significant differences in hematological and biochemical variables.	[82]
Acute diarrhea (AD)	66	7 days	*Lactobacillus fermentum* VET9A, *L. rhamnosus* VET16A, and *L. plantarum* VET14A	Significantly decreased the pathogenic bacteria such as *Escherichia coli* and *Clostridium perfringens.*	No data.	Significantly improved stool consistency. Reduced vomiting.	There were no significant differences in hematological and biochemical variables.	[36]
Acute diarrhea (AD)	36	8 days	*Lactobacillus farciminis* (porcine origin)*, Pediococcus acidilactici* (unknown origin)*, Bacillus subtilis* (soil origin), *Bacillus licheniformis* (soil origin), and thermo-stabilised *Lactobacillus acidophilus* MA 64/4E (human origin)	No data.	No data.	Significantly improved the condition of stool but not the vomiting episodes.	No data.	[84]
Acute diarrhea (AD)	60	20days	*Bifidobacterium bifidum* VPBB-6, *Bifidobacterium longum* VPBL-5, *Bifidobacterium animalis* VPBA-4, *Bifidobacterium infantis* VPBI-6, *Lactobacillus acidophilus* VPLA-4, *Lactobacillus plantarum* VPLP-5, *Lactobacillus casei* VPLC-1, *Lactobacillus brevis* VPLB-5, *Lactobacillus reuteri* VPLR-1, and *Lactobacillus bulgaricus* VPLB-7	No significant data showed that the number of pathogenic bacteria decreased.	No data.	The days of diarrhea achieved acceptable fecal consistency, shortened but not significant.	No data.	[87]
Acute hemorrhagic diarrhea syndrome (AHDS)	25	21 days	*Lactobacillus plantarum* DSM 24730, *Streptococcus thermophilus* DSM 24731, *Bifidobacterium breve* DSM 24732, *Lactobacillus paracasei* DSM *24733*, *Lactobacillus delbrueckii* DSM 24734, *Lactobacillus acidophilus* DSM 24735, *Bifidobacterium longum* 120 DSM 24736, and *Bifidobacterium infantis* DSM 24737	*Blautia and Faecalibacterium* significantly increased and *Clostridium perfringens* significantly decreased.	No data.	Significantly improved clinical condition while the CHDSI did not show significant difference compared to placebo group.	There were no significant differences in hematological and biochemical variables.	[35]
Chronic inflammatory enteropathies (CIE)	14	30 days	*Ascophyllum nodosum* and *Bacillus subtilis* C-3102	The *Prevotella* genus increased but not significantly.	No data.	The CIBDAI did not significantly change.	Significantly upregulated the concentrations of acetate, isovalerate, and isobutyrate.	[88]
Chronic enteropathies (CE)	20	42 days	*Lactobacillus acidophilus‚ Lactobacillus casei‚ Enterococcus faecium,* and *Bacillus subtilis*	*Clostridium* spp. and *Bacteroides* spp., which produce SCFAs, significantly increased. *Enterobacteriaceae* significantly decreased.	No data.	There was no significant difference in clinical, endoscopic, and CCECAI results.	Serum levels of hs-CRP significantly decreased.	[92]
Inflammatory Bowel Disease (IBD)	20	60 days	*Lactobacillus* (*L. casei*, *L. plantarum*, *L. acidophilus*, *L. delbrueckii*, and *L. bulgaricus*) and *Bifidobacterium* (*B. longum*, *B. breve*, and *B. infantis*) *thermophilus*	*Faecalibacterium* spp. significantly increased.	FoxP3^+^ cells significantly increased CD3^+^ T-cells significantly decreased, and TGF-β^+^ was significantly more expressed.	There were no significant difference in CIBDAI score and histology score in either group.	No data.	[93]
Gastroenteritis	120	7 days	*Lactobacillus johnsonii* CRL1693, *Ligilactobacillus murinus* CRL1695, *Limosilactobacillus mucosae* CRL1696c, and *Ligilactobacillus salivarius*	Enterobacteria and enterococci decreased but not significantly.	No data.	The consistency of stool significantly improved.	No data.	[94]

Note: CIBDAI: canine IBD activity index; CCEAI: canine chronic enteropathy clinical activity index; CHDSI: canine hemorrhagic diarrhea severity index; hs-CRP: high-sensitivity C-reactive protein.

## Data Availability

This manuscript is based on a review of existing literature analysis and does not include any new datasets or materials. All sources used are cited appropriately within the article.

## Abbreviations

IBD: inflammatory bowel disease; AHD: acute hemorrhagic diarrhea; AD: acute diarrhea; FAO: Food and Agriculture Organization of the United Nations; WHO: World Health Organization; ISAPP: International Scientific Association for Probiotics and Prebiotics; SCFAs: short-chain fatty acids; MOS: mannose-oligosaccharides; FOS: fructo-oligosaccharides; GOS: galacto-oligosaccharides; GPCRs: G-protein coupled receptors; TFs: transcription factors; AMPs: antimicrobial peptides; HDAC: histone deacetylase; TLRs: Toll-like receptors; NLRs: NOD-like receptors; CCECAI: Canine Chronic Enteropathy Clinical Activity Index; CIBDAI: Canine inflammatory bowel disease index; ESPGHAN: European Society for Paediatric Gastroenterology, Hepatology, and Nutrition; CCEAI: canine chronic enteropathy clinical activity index; CHDSI: canine hemorrhagic diarrhea severity index; hs-CRP: high-sensitivity C-reactive protein.
